# Multimodal imaging of tibialis anterior muscle adaptations to neutral-position immobilization

**DOI:** 10.1371/journal.pone.0339510

**Published:** 2026-01-30

**Authors:** Sheiren A. Martínez-Méndez, Berenice Martínez-Gutierrez, Zuriel Casillas-Marquez, Bertha Segura-Alegria, Iván Rosado-Méndez, Carla P. Villanueva-Meléndez, Ismael Jimenez-Estrada, Karla García-Pelagio

**Affiliations:** 1 Departamento de Física, Facultad de Ciencias, Universidad Nacional Autónoma de México, Ciudad de México, México; 2 Laboratorio de Mecanobiología del Músculo, Facultad de Ciencias, Universidad Nacional Autónoma de México, Ciudad de México, México; 3 Departamento de Biología, FES Iztacala, Universidad Nacional Autónoma de México, Tlalnepantla, México; 4 Department of Medical Physics, University of Wisconsin-Madison, United States of America; 5 ENES-León, Universidad Nacional Autónoma de México, Guanajuato, México; 6 Departamento de Fisiología, Biofísica y Neurociencias, Centro de Investigación y Estudios Avanzados del IPN, Ciudad de México, México; Lorestan University, IRAN, ISLAMIC REPUBLIC OF

## Abstract

Muscle disuse atrophy is a frequent consequence of therapeutic immobilization following sport injuries, bone fractures, and ligament tears, often resulting in marked reduction of muscle volume, mass, and strength. Despite the widespread use of neutral-position limb immobilization in clinical practice, its physiological effects remain insufficiently characterized. To address this gap, we employed thermographic, tomographic, and ultrasound imaging to assess how neutral-position immobilization (Imm) affects the tibialis anterior, a predominantly fast-twitch ankle dorsiflexor muscle that plays a key role in foot deceleration after heel strike, provides functional stability during gait preventing falls and contributes substantially to load absorption, in twenty-seven young male Wistar rats after 7 and 14 days of treatment. To complement these, force measurements and histology were analyzed. Our results showed a significant limb temperature increase of up to 10% after 14 days compared to controls accompanied by a volume reduction of 38% (p < 0.05) confirmed by tomography and a 2-fold (p < 0.05) increment of CNFs denoted by histology (H&E). At 14 days of Imm ultrasound imaging highlighted changes in subcutaneous tissue thickness, and increased connective tissue; a significant 2-fold reduction in specific force during muscle twitch and 28% (p < 0.05) in tetany. Fiber type conversion mainly to type IIA (intermediate) was evident on histology and supported by the prolonged fatigue time following two fatigue protocols (continuous stimulation and repeated short-tetany) for up to 50% (p < 0.05) after 14 d of Imm. Our results demonstrate that, although immobilization in a neutral position is the best practice in the clinic, it carries important detrimental changes in muscle structure and physiology. These findings underscore the importance of integrating clinical imaging techniques to monitor muscle status during immobilization and rehabilitation, enabling more effective and timely interventions.

## Introduction

Immobilizing the hind limb in a neutral position which closely mimics clinical scenarios after an injury provides protection, minimizes further damage, promotes proper healing, and prepares for rehabilitation. Deviations from not immobilizing in the neutral position (dorsi- or plantar-flexion) can introduce adverse problems such as altered muscle tension, joint capsule strain, uneven vascular perfusion, differential nerve compression, among others [1,2]. It is a foundational part of managing injuries to ensure that the limb recovers in a way that restores its entire function and prevents long-term complications. Although *per se* immobilization causes protein degradation, synthesis alteration, loss of muscle mass and strength causing impaired motility that could end in both a decline of the basal metabolic rate and in cardiovascular problems, i.e., muscular atrophy [1,3,4], immobilizing is still a good option after an injury. So immobilizing in an appropriate position such as in the neutral one, is important as it ensures that the joint, bones, and soft tissue are aligned in their natural, stable state preserving its normal range of motion; reduces muscle contractures and the risk of overstretching or damaging the ligaments, or other surrounding muscles [1].

Physical examination is useful for monitoring healing, although visualizing the hind limb using imaging techniques such as infrared imaging, ultrasound, tomography or magnetic resonance after immobilization is primordial to ensure proper positioning, identifying complications, and adjusting care plans.

Thermography or infrared imaging (IR) is a non-invasive affordable indirect method to monitor temperature variations that can indicate underlying physiological and vascular changes derived by immobilization. It can help identify heat patterns caused by increased blood flow as a signal of recovery and healing process, or swelling and inflammation in the hind limb as a deleterious effect [5]. This technique has been used from inflammatory diagnose lesions to circulatory and microcirculatory alterations giving a good approach of what is happening [6,7].

Computer tomography (CT) provide high-resolution imaging, allowing precise visualization of bones, muscles, tendons, and joints specially if a contrast agent is used [8]. The radiation dose is less compared to Photon Emission Tomography (PET) or Single Photon Emission Computer Tomography (SPECT) but higher than conventional X-rays. CT is especially valuable for assessing the degree of muscle atrophy showed as a reduction in the cross-sectional area of the muscles or fatty infiltration into the muscle tissue, which might be slightly visible as changes in density. CT is particularly effective in evaluating bone structures, detecting stress fractures, and assessing bone density [9]. It is worthy to mention that the European Working Group on Sarcopenia in Older People (EWGSOP) considers CT and MR gold standard methods for the evaluation of muscle mass.

Ultrasound (US) as thermography is another non-invasive easily accessible portable device for monitoring muscle atrophy, inflammation, soft tissue changes, tendon and ligament health and vascular integrity with high-resolution images. It can show muscle thickness, which may decrease after immobilization, or even identifying fluid accumulation or signs of inflammation denoted by hypoechoic areas (darker areas in the image, indicating fluid) [10].

Despite being the gold standard for assessing muscle quality and quantity, MRI remains limited in clinical practice due to its high cost, limited accessibility, lengthy examination times, and the need for highly trained personnel [11].

Several classic experiments have studied the physiological changes of ankle plantar flexors muscles after an atrophic immobilized condition in dorsi flexion or plantar flexion [12–17] position, but studying the strongest ankle dorsiflexor muscle, the tibialis anterior (TA) in neutral position has not gained enough importance [16]. The tibialis anterior (TA) muscle plays a critical role in positioning the foot in space and in maintaining balance and functional stability during gait [18]. It is particularly essential during foot deceleration following heel strike, where it contributes substantially to load absorption [19]. Moreover, by ensuring adequate foot clearance during the swing phase, the TA reduces the risk of falls [20,21]. Consequently, TA hypotrophy or inactivation can lead to severe functional limitations that compromise patient independence. A comprehensive understanding of its mechanics and behavior under conditions of pronounced atrophy is therefore vital for the development of effective rehabilitation strategies for individuals who have undergone prolonged immobilization or extended periods of bed rest. For this, maintaining the ankle joint in a neutral tibio-talar position during immobilization is essential, as it supports an intermediate muscle length and reduces the likelihood of chronic structural alterations [22]. This neutral alignment is also mechanically advantageous: in the event that complications such as fibrosis, capsular contracture, or ankylosis arise, foot function is more likely to be preserved, thereby supporting efficient gait with reduced compensatory demands [23]. By characterizing the physiological consequences of immobilization in a neutral position, an approach that more closely reflects standard clinical practice, we aim to advance the understanding of muscle responses under conditions that approximate real-world therapeutic immobilization. Accordingly, the goal of this study is to determine whether thermography, tomography, and ultrasound imaging modalities can yield meaningful insights into alterations in contractile properties and fatigue of the tibialis anterior (TA) muscle in male rats following hind limb immobilization in a neutral position.

## Materials and methods

### Animals

Twenty-seven young Wistar male rats (270 ± 20 g) provided by the UNAM-Faculty of Sciences Animal Facility were used for all studies reported here. All of our protocols were approved by the Institutional Animal Care and Use Committee at Faculty of Sciences-UNAM following the guidelines of the Mexican Official Norm (NOM-062-ZOO-1999). Rats were maintained on standard chow with *ad libitum* access to water and housed under a 12-h light–dark cycle. For terminal procedures, animals were euthanized using an overdose of inhaled isoflurane. All experimental procedures were conducted in accordance with the ARRIVE guidelines.

### Cast immobilization

For the immobilization protocol, animals were initially anesthetized with 4–5% isoflurane and subsequently maintained at 2% isoflurane in oxygen delivered at 0.8 L/min. Once a stable and adequate depth of anesthesia was confirmed, the experimental procedures were initiated. During the procedure, the animals were kept warm (24.0 ± 0.5°C) by the use of a heat lamp. Saline solution was applied to each eye to protect the corneas from drying. The rats were divided into three groups: 1) control rats with no hind limb immobilized (C or 0d; n = 9), and rats with its right hind limb (Imm) immobilized 2) for 7 days (7d; n = 9) or 3) for 14 days (14d; n = 9).

Under anesthesia, adhesive spray (Q.D.A. Spray, Cramer, USA) was applied to the right limb followed by under-wrapped strips from distal to proximal below the pubis to hold back the fur and to protect the skin. Then, a modified Thomas splint consisting of two metal shafts in U-shape was made of 22-gauge copper wire and adjusted to each animal from the phalanges to the middle part of the femur, assuring a proper fit [24]. Finally, a plaster bandage was fixed to the hind limb, placing the toe in a neutral natural position. Before returning the animal to its cage, a 10–15 min period was needed to let the plaster dry. Animals were monitored constantly for inflammation, swelling edema, or problems with ambulation. If any signs of deterioration were observed, the cast was removed immediately. The animals were allowed to eat and drink *ad libitum* and free to move using their left hind limb and both fore limbs.

### Infrared thermography

**Fig 1:**
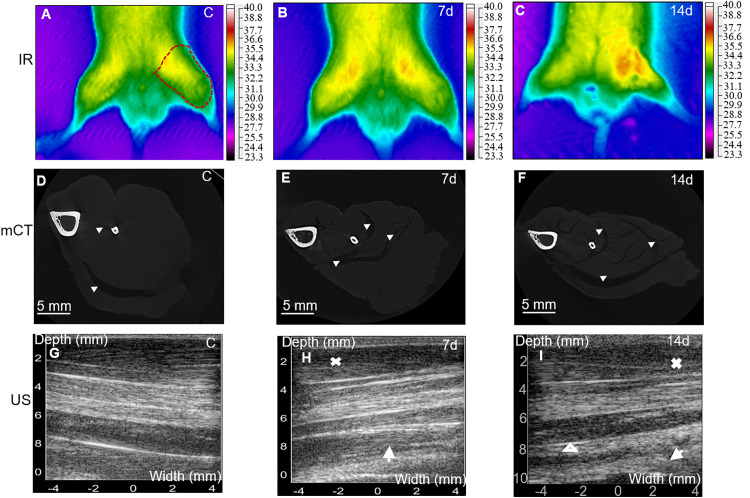
Representative thermographic, tomographic and ultrasound images of hind limb and TA muscle at 0, 7 and 14 days of immobilization. IR thermography was acquired with the anesthetized animal in supine position indicating a slight increment in temperature in the Imm extremities compared to the C one due to a regenerative ongoing process. Radiant heat color coding (23.3 - 40.0 °C) is shown to the right of each image (n = 5) **(A-C)**. Micro-CT transversal images show soft tissue (perimuscular adipose tissue, TA, extensor digitorum longus, extensor halluces longus, gastrocnemius and soleus) and the bones (tibia and fibula). Arrowheads denotes the striation pattern. (n = 3) **(D-F)**. Ultrasound longitudinal views at 7d and 14d denoted areas of hypo-echogenicity (arrows) followed by an increment in subcutaneous area (crosses), and connective, fibrous and/or fatty tissue (arrow heads) (n = 3) (G-I).

A calibrated infrared (IR) camera (Fluke TiS60 + , USA) using an emissivity point of 0.98 ± 0.05, thermal sensitivity of <30 mK and frequency acquisition rate of 60 Hz was used within a range of 20–40°C. The experimental room was set at 20.0 ± 0.5°C, ensuring the absence of detectable air currents; if present, only laminar airflow was permitted to avoid thermal disturbance. Uniform LED lighting was used to minimize infrared interference. Once the immobilization time passed, the cast was removed under anesthesia, and the animal was positioned supine on a polished aluminum table (a low-emissivity material selected to reduce thermal artifacts). The limbs were gently secured with tape for 15 min to allow temperature stabilization and to eliminate thermal noise associated with handling. Temperature was assessed by averaging two thermographic images at 0, 7 and 14 days of Imm covering the thigh-to-lower-leg region, including the tibia–fibula muscle compartment (red segment in Fig 1A), using SmartView software (Fluke v4.4) with a 20–40 °C high-contrast palette positioning the camera 30 ± 1 cm perpendicular to the animal.

### Micro-CT images

Once the IRR and US studies were performed, immobilized limbs at all-time points were removed from the animal. The fur was completely pulled out from the insertion of the head of the femur to the toe assuring that the limb musculature remains intact. Then, under *ex vivo* conditions limbs were placed in a bath of 100% ethanol for 14 days a procedure that allows high-resolution morphological visualization of hind limb structures, as previously reported by our laboratory [24]. Briefly, once the bath time finished, individual hind limbs were placed on a dry gauze and left at room temperature for 20–25 min to allow the ethanol to evaporate, then scanned inside a 50 ml Eppendorf tube. Hind limb images were acquired with no contrast agent using a micro-CT (SkyScan 1276, Bruker, Germany) calibrated to water. The calibration is needed as standard reference point for converting arbitrary X-ray attenuation values into standardized units, either Hounsfield Units (HU) or physical material density. The parameters for the acquisition were 40 kV, 150 µA, no filter, step and shoot mode, rotation step of 0.4 °, pixel size of 14 µm and 4018 x 2016 matrix. Reconstruction and image analysis was done with CTan software (Bruker, v 1.19). The obtained raw images were displayed in a palette of 255 channels representing gray intensity levels which depend on the X-ray attenuation values of the tissue. A volume of interest of 20 slices equivalent to 2 mm was delineated on each acquired image by placing a 250-pixel circular region of interest (ROI) in the broader portion of the hind limb axial plane having the TA and the tibial tuberosity as anatomical references. Linear attenuation coefficient (µ, a measure of how much a material reduces the intensity of X-ray per unit thickness reflected as gray intensities) was calculated three times [25] in each ROI using DataViewer software (Bruker v. 1.5). A grayscale scoring system utilizing Image J (NIH, v 1.53) based on grayscale intensities which is directly proportional to µ was used to identify and quantify muscle (41–71), bone (72–255) and the striation patterns (0–40), indicator of muscle degeneration [24]. The use of organic solvent–based fixation, such as alcohol, induces protein denaturation by removing water from free carboxyl, hydroxyl, amino, amido, and imino groups, which in turn leads to tissue shrinkage [26] and subsequent reductions in muscle mass, muscle volume, and fiber cross-sectional area [27,28]. Because all samples in this study were subjected to the same alcohol-based fixation method, the extent of tissue shrinkage was comparable between control and immobilized groups. Consequently, changes in striation area fraction were used as an index of muscle degeneration (Fig 1D–F).

### Ultrasound

High-frequency B-mode ultrasound images of C and Imm TA muscles were obtained to evaluate structural indicators of muscle damage with a Verasonics Vantage 128 system (Verasonics Inc., USA) using the L35-16vX transducer operated at 25 MHz enabling high-resolution visualization of superficial skeletal muscle architecture. Images were taken 1.5 hrs after cast removal [10]. Prior to imaging, the hind limb was shaved to enhance acoustic coupling and improve delineation of fascicular structures. Under anesthesia, the animal was placed in a supine position on a temperature-controlled heating pad (30–32 °C). The hind limb was gently externally rotated, and the ankle was secured in a neutral position with tape to ensure consistent alignment across subjects. Longitudinal views of the TA muscle were obtained by placing the transducer along the anterior compartment of both hind limbs midway between the inferior pole of the patella and the lateral malleolus. This orientation enables reliable visualization of muscle fascicles, thereby facilitating the detection of dynamic retractions (indicative of muscle shortening) as well as static abnormalities such as contractures. Raw in-phase and quadrature (I/Q) data were collected directly from the system and subsequently processed to reconstruct radiofrequency (RF) echo signals via modulation at the transducer’s center frequency. B-mode images were generated by computing the logarithm of the Hilbert transform of the RF data, producing conventional brightness-mode representations of muscle architecture. To reduce speckle noise while preserving structural detail, a 3 × 3-pixel Gaussian spatial filter was applied to the final images.

### Muscle force measurements

**Fig 2:**
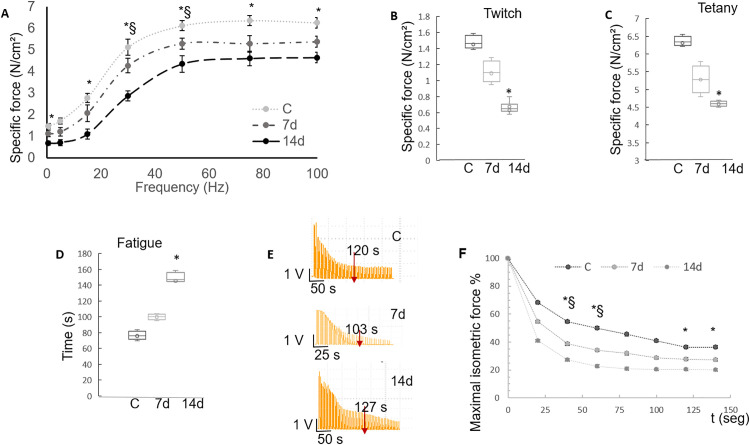
Measurements of contractile force (A-C) and fatigability (D-F). Force-frequency relationship for 0d, 7d and 14d of Imm. Difference between C and 7d is significant at 30 and 50 Hz, while at 14 d all the frequencies were significant except at 5 Hz (S2 Table) (A). Single twitch and maximal tetanic force of TA muscle were measured in situ at 0,7 and 14 d of Imm. At 14d of immobilization yields a significant decrease in twitch and tetanic force (S1 Table) (B, C). Continuous stimulation fatigue protocol. Time passed for the TA muscle to reach a 20% of its force of that obtained in the first stimuli showed that the TA muscle immobilized at 14d reached its fatigue in a double time compared to control (S3 Table) (D). Representative trace recordings of fatigue at maximal isometric force in TA muscles in a repeated short tetany protocol fatigue (S3 Table) (E). In situ assays showed increased fatigue at 2 minutes in both the 7-day and 14-day groups (F). All data are presented as means ± SD. § represents a significant difference of p < 0.05 for 7 days and * represents a significant difference of p < 0.05 for 14 days of immobilization. (n = 3).

Contractile function of immobilized and control TA muscles (n *=* 3 per group) was measured as described previously [29–31]. While the animals remained under anesthesia, right TA muscle was prepared for *in situ* contractile testing. One tendon was left intact at its insertion near the knee, whereas the distal tendon was secured to a force transducer (Grass FT03B, USA). Force calibration was performed by generating a mass–voltage calibration curve, in which known masses were sequentially hung to the force transducer to establish the linear relationship between applied load and output voltage; as expected, increasing mass produced proportionally higher voltage readings, allowing conversion of raw voltage signals into absolute force values. Experiment was done by triplicate. Muscle activation was elicited using rectangular current pulses (1 ms duration, 1 Hz frequency) delivered through a pair of silver electrodes connected to a stimulator (Grass SD9, USA). Single-twitch responses were first obtained at progressively varied muscle lengths to identify the optimal length (Lo) at which maximal twitch force was produced. Following determination of Lo, the TA was subjected to a series of three isometric twitches every 2 s at increasing stimulation frequencies (0.5, 5, 15, 30, 50, 75, and 100 Hz) to construct a force–frequency relationship (Fig 2A). A single tetanic contraction was then evoked using high-frequency (75 Hz) and 5 mA supramaximal current pulses. Once the force-frequency was obtained, two fatigue protocols were performed [32]. 1) Continuous stimulation: The muscle underwent uninterrupted supramaximal stimulation at 75 Hz Fatigue was quantified as the time required for peak force (Po) to decline to 20% of its initial value. To verify muscle viability after the fatiguing bout, the muscle was allowed to recover for 5 min, after which a 30 Hz stimulation was applied. If the force generated after recovery was less than 10% from the pre-fatigue force, the experiment was viable; otherwise, the trial was discarded. After a 30-min recovery period, the second protocol was initiated. 2) Repeated short-tetany: Each trial began with a 30 Hz reference pulse, followed by supramaximal 75 Hz tetanic trains lasting 2 s, separated by 3 s rest intervals (Fig 2E–F). Stimulation continued until Po declined to 20% of its initial value and fatigue was then calculated as the elapsed time between the initial Po and the point at which Po reached 20%. Muscle viability was reassessed giving a 30 Hz stimulation and only if force recovery remained within 10% of the reference pulse, the experiment was accepted.

Stress or specific force is defined by [N/cm^2^] = F/CSA where F is the force (N) normalized by the muscle cross-section area (CSA) (cm^2^). CSA = (muscle mass[g])/ (length at L_o_ [cm]) * (muscle density [g/cm^3^]); where muscle mass is the wet weight of the muscle; L_o_ is the optimal muscle length; and muscle density is assumed to be a constant (1.067 g/cm^3^) [30].

### Hematoxylin and eosin (H&E) staining

Cross-sections of 10–14 μm in thickness were obtained from the midbelly region of unfixed, snap-frozen right TA muscles from the hind limb of control and immobilized animals. After TA removal it was immediately thaw in liquid nitrogen and stored at −80 °C. Then tissue samples were cryosectioned at −20 °C and fixed in cold acetone to immobilize cellular components and enhance dye penetration, and then allowed to air-dry. Standard hematoxylin–eosin staining protocol was used to visualize muscle fiber morphology. Sections were immersed in Harris hematoxylin (Sigma-Aldrich, USA) for 3 min to stain nuclei and other basophilic structures, followed by thorough rinsing in room-temperature tap water to remove excess dye and promote proper nuclear definition. Then, dipped three times in Wright eosin (Sigma-Aldrich, USA) to stain cytoplasmic and myofibrillar components. Slides were subsequently transferred to 95% ethanol to dehydrate the tissue and stabilize the stain. This protocol allowed reliable analysis of muscle fiber morphology, cross-sectional area, and structural alterations in both control and immobilized samples.

### Nicotinamide adenine dinucleotide tetrazolium reductase (NADH-TR) staining

After cross-sectioning the TA midbelly, 10–14 μm thick sections were incubated for 1hr at 37°C in a 1:1 nitroblue tetrazolium- Nicotinamide adenine dinucleotide solution (NADH-TR) (NBT diluted in 50 mM tris buffer, pH 7.6; 2.25 mM mixed with NADH diluted in 50 mM tris buffer; Sigma-Aldrich). Following incubation, sections were rinsed three times with distilled water to halt the enzymatic reaction. Samples were then placed 3-times through a gradient of increasing concentrations of acetone (30%, 60% and 90%) for NBT removal [33]. This technique distinguishes glycolytic or oxidative/glycolytic fast-fatiguing fibers (light purple) from oxidative, slow, fatigue-resistant fibers (intense purple), thereby enabling detailed analysis of fiber-type composition.

### ATPase staining

10–14 μm thick cryosections were processed for alkaline myosin ATPase (pH 9.4) an established method for distinguished muscle fiber subtypes baed on enzymatic activity. Sections were pre-incubated for 20 minutes at 37 °C in a TRIS buffer (HCl/CaCl₂; Sigma-Aldrich, MO, USA) adjusted to pH 9.4 to condition the myofibrillar ATPase and enhance pH-dependent differentiation. After pre-incubation, the slides were rinsed 15 times with distilled water to remove residual buffer components. A second incubation was then performed using the working solution—TRIS HCl/CaCl₂ buffer supplemented with ATP (Sigma-Aldrich)—for 60 minutes at 37 °C, allowing myosin ATPase hydrolysis to occur selectively within distinct fiber populations. Then washed 15 times with distilled water and subjected to a second incubation in a working buffer (TRIS HCl/CaCl, ATP) (Sigma Aldrich, MO, USA) at pH 9.4 for 60 minutes at 37 °C. After incubation, samples were washed 15 times with distilled water, then immersed in 2% CaCl for 3 minutes, continued by another immersion in 2% CoCl for 3 minutes, and washed 20 times with distilled water. Finally, the samples were left in 10% (NH₄)₂S for 3–5 seconds which produced a dark, stable coloration indicative of ATPase activity [33]. This technique allows differentiation between fast-, intermediate-, and slow-twitch muscle fibers based on the pH-activity of myosin ATPase, an enzyme activated by Ca^2^ ⁺ that hydrolyzes ATP to form ADP during muscle contraction [1].

For histology, all tissue sections were mounted on coverslips with Permount mounting medium (Thermo Fisher Scientific, USA). The stained sections were examined using a bright field light microscope (Zeiss Axioscope, Carl Zeiss, Germany) equipped with either a 10× or 20 × objective in combination with a 10 × eyepiece. For each muscle, digital micrographs were acquired from seven randomly selected, non-overlapping fields spanning the mid-belly region of the section, with three samples analyzed per group (Control and Immobilized). These images were subsequently used to quantify minimal Feret’s diameter, the proportion of centrally nucleated fibers (CNFs), and muscle fiber-type composition following established protocols [30]. All analyses were performed using ImageJ software (version 1.52, NIH, USA).

### Statistics

All values were reported as mean ± SD. Statistical significance was assessed with independent Student’s *t* and one-way ANOVA tests with a Tukey’s posthoc analysis. For fiber diameter, Fischer’s exact test and χ^2^-determinants was used. *P < 0.05 was considered statistically significant.

## Results

Immobilization in a neutral position of a hind limb after an injury is the best procedure to ensure proper healing although this can have detrimental consequences being the most important, muscular atrophy for disuse, which can alter the structure and physiology of the muscle.

Firstly, after immobilization we observed a slight but significant gain in the animal body weight of 6% (p < 0.05) and 9% (p < 0.05) at 7 and 14 days of immobilization, respectively, attributed to the restriction of physical activity, see Table 1. While TA mass and muscle diameter at 14 d in the immobilized TA decreased 11% (p < 0.05) and 15% (p < 0.05), respectively. This goes by an 11% (p < 0.05) and 23% (p < 0.05) reduction in muscle area fraction at 7 and 14 d of Imm, respectively (Table 1, Fig 1E, F). As muscle atrophy progressed, the striated appearance of the tissue became increasingly pronounced (up to 22%, p < 0.05; Fig 1E, F). The TA weight–to–body weight ratio normalizes muscle mass to account for individual differences in animal size. At 14 days of immobilization, this ratio decreased to 1.91 (p < 0.05), indicating a significant reduction in TA mass compared with the control group (2.38).

**Table 1:** Morphological and Physiological changes in Immobilized hind limb muscle.

	Control	7d	14d
Animal body weight (g)	259.6 ± 19.1	277.2 ± 41.2 *	286.5 ± 42.4 *°
**Hind limb characterization**			
Temperature (°C)	33.67 ± 0.41	34.17 ± 0.34	37.44 ± 0.55 *°
Muscle Area fraction reduction (%)		11.3%	23.4% °
Striations appearance (%)	3.9%	10.0% *	22.5% *°
Muscle volume in a 2 mm section (mm³)	1.67 ± 0.12	1.19 ± 0.09 *	1.03 ± 0.11 *
Ratio TA weight/body weight (x10¯³)	2.38 ± 0.26	2.02 ± 0.24	1.91 ± 0.21 *
**Tibialis Anterior characterization**			
Weight (g)	0.62 ± 0.05	0.56 ± 0.10	0.55 ± 0.09*
Diameter (cm)	1.03 ± 0.08	0.81 ± 0.01 *	0.87 ± 0.11 *
Length (cm)	2.53 ± 0.21	2.56 ± 0.16	2.55 ± 0.32
Ratio TA weight/Diameter	0.60	0.69	0.63
Centrally nucleated fibers (CNFs) (%)	0.89 ± 0.1	1.37 ± 0.1 *	2.03 ± 0.2 *°
Specific twitch force (mN/mg)	1.47 ± 0.13	1.12 ± 0.45	0.67 ± 0.20 *°
Specific tetanic force (mN/mg)	6.35 ± 0.31	5.28 ± 1.50	4.59 ± 1.07 *
Repeated short tetany Fatigue (s)	120 ± 7.17	103 ± 15.99	127 ± 18.73

Values are means ± SD. *p < 0.05 represents a statistical difference compared to control. ° p *<* 0.05 represents a statistical difference between experimental groups.

**Fig 3:**
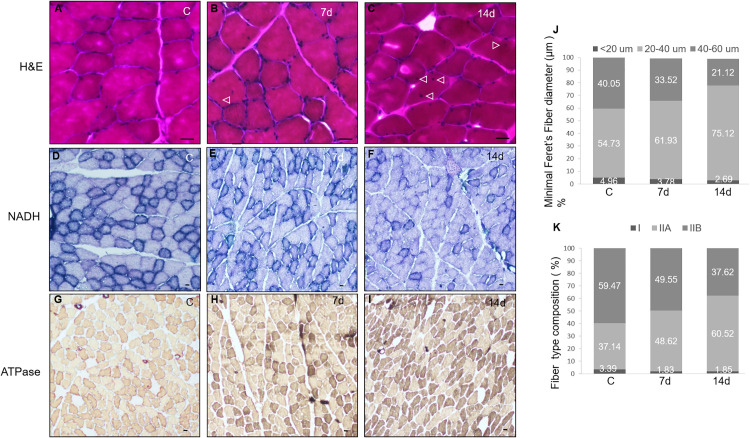
Cryosections of TA fibers stained with H&E (A-C), NADH (D-F) and ATPase (G-I) showed morphometric changes at 7d and 14d after Imm. In H&E the myoplasm is stained in pink and nuclei in dark blue revealing a reduction in fiber’s diameter accompanied by the presence of central nuclei (arrow) at 7 and 14 days (B, C). NADH staining showed an increment of light-purple fibers corresponding to fast-oxidative (type IIa) and fast-glucolytic (IIb) fibers at 7 (E) and in a higher percentage at 14 days (F) of Imm opposite to the reduced amount of dark purple fibers represented by the slow fibers (D). ATPase technique showed an increment of IIA (dark-brown) and IIB (light-brown) fibers at 7 and 14 d (H, I) compared to control which has the higher amount of type I fibers (white color) (G). The TA fiber diameter was quantified from H&E sections at all time points and presented as a histogram using the minimal Feret’s diameter (J). Fiber-type composition—classified as slow, fast oxidative, and fast glycolytic fibers—was determined from ATPase-stained sections and summarized in a histogram (K). Scale bars = 15 µm from A-E and 20 µm from D-I. (n = 3).

Afterwards, muscular atrophy signs were also detected with three imaging techniques, IR, CT and US (Fig. 1) which gave us insight of the macroscopic physiological TA changes. Thermograms were obtained in supine position to image a compound of muscles which includes: vastus lateralis, rectus femoris, tibial anterior and quadriceps at 0, 7 and 14 d of immobilization (Fig 1A-C). The temperature increment is related to the duration of immobilization, so the highest increase in temperature throughout the entire hind limb was of 11% (p < 0.05) at 14d compared to C. After 7 days, the TA showed a mild increase in temperature, although not significant, due to early inflammation and circulatory changes. By 14 days, the healing response is more advanced, and the observed temperature increase reflects heightened inflammation, enhanced blood flow, and ongoing muscle regeneration. So, the healing process after 14 days of immobilization caused an increase in temperature compared to the healing process after just 7 days, as the tissue undergoes more pronounced metabolic activity and repair process. Rise in temperature is consistent with a higher number of CNFs in TA (2% at 14d; p < 0.05; Table 1, Fig 3 B, C) due to regeneration process [34].

Tomographic images of a cross-sectional region of the anterior compartment were analyzed denoting a 10% (p < 0.05) volume decrement at 7d and 40% at 14d (p < 0.05). Appearance of dark areas, so called striations, within the soft tissues (muscle fat and connective tissue) representing air and residual perimuscular and intramuscular adipose tissue or even intramyocellular lipid (IMCL) droplets in smaller quantities reached its peak at 14d by 22% (p < 0.05) followed by 10% at 7d (p < 0.05) compared to control with just 4% (Table 1). The observed volume reduction in the Imm hind limb translate in strength decrement (Fig 2A-C), changes in fatigue resistance (Fig 2 E, F) and in fiber diameter reduction (Fig 3B,C and J).

Ultrasound images can detect muscle adaptations based on the changes in muscle echogenicity [10]. Thickness of subcutaneous tissue (observed as an accumulation of lipid tissue between the skin and muscle tissue) was observed at 7 and 14 days, which is paralleled by the appearance of hypo-echogenic bands close to the cortical bone (Fig. 1 H, I). Alterations in depth and hypo-echogenic areas were associated with changes in muscle tissue composition by lipid reserves and connective tissue increment leading to a reduction in the number of fibers and of serial sarcomeres accompanied by accumulation of connective tissue in the perimysium at 7 and 14 days. These alterations resulted in a measurable reduction in muscle volume and a disruption of normal muscle architecture (Fig 1E–F, Fig 3B–C), ultimately contributing to impaired muscle function in immobilized animals (Table 1). The decline in force production observed with prolonged immobilization (Fig 2A) was consistent with the emergence of inhomogeneous fibrillar regions (Fig 1H–I).

We evaluated the functional changes evoked in immobilized muscles by measuring the maximum isometric contractile force (P_o_) produced by TA muscles in anesthetized animals. Single twitch force of the TA muscle was 2.2 times lower as the immobilization time increased (Table 1). Interestingly, specific tetanic force (75 Hz stimulation) of Imm TA muscles showed a reduction of 28% at 14d of Imm respect to control (p < 0.05; Table 1, Fig 2 A, C, S1 Table). *P*_*o*_ vs stimulation frequency is depicted in Fig 2A and S2 Table. For 7 and 14 of Imm, *P*_*o*_ has a lower tendency at all frequencies, particularly at 14d where differences reached 35% at 100 Hz (p < 0.05). This result suggests that force production in immobilized fast-twitch muscles decreased markedly at early time points. In addition, we also tested the rate of muscle fatigue *in situ* following two fatigue protocols (see Methods section). Fatigue induced by the continuous stimulation protocol increased the time to fatigue for up 2-fold (p ≤ 0.05) in immobilized muscles for 14 days compared to controls (Fig 2 D, S3 Table). While fatigue induced by short tetanic stimuli showed an increase in the time to fatigue of up to 5% at 14 days of immobilization (Fig 2 E,F; S3 Table) although not statistically significant.

For studying microscopic muscular changes, morphology and muscle structure were studied to assess the amount of atrophy after the immobilization. Using the H&E technique, it was found that the mean minimal Feret’s diameter was smaller at all immobilization times (Fig 3 B, C) compared to controls. A marker of ongoing degeneration and regeneration in adult skeletal muscle is the number of central nuclei fibers (CNFs) observed at all-time points (0.9%, 1.3% and 2.0% for 0d, 7d and 14d, respectively, p < 0.05; Table 1, Fig 3 A-C). NADH was used to detect the activity of NADH-TR, which is an oxidoreductase enzyme present in the mitochondria of skeletal muscle fibers. Type I fibers appear more intensely stained compared to type II fibers because their content of mitochondria is higher and, therefore, exhibit higher oxidative activity having an increment of type II fibers at 7 and 14d of Imm (Fig 3 D-F), although the uneven color of the stain across the myofiber makes NADH staining problematic to distinguished between intermediate and fast fibers detection. ATPase staining can be used for fiber typing because of the strong contrast between stained and unstained myofibers (Fig 3 G-I) showing an increment of intermediate fibers in the immobilized hind limb of 38% or 23% at 7d and 14d respectively compared to control which explains the fatigue resistance at 14d (Fig 2 D). Data suggests that immobilization resulted in a conversion of oxidative slow-twitch and glycolytic fast-twitch muscle fibers into intermediate-twitch muscle fibers. Force production and fatigue results are paralleled with findings in fiber type conversion to intermediate ones. Since our results are independent on muscle weight (specific force), the apparent weakness of immobilized muscles could be attributed to morphological, metabolic and fiber type composition changes (Table 1, Fig 3 D-I, K).

## Discussion

In clinical practice, prolonged hind limb immobilization following injury is a well-established model that induces skeletal muscle disuse atrophy. The absence of mechanical loading leads to a rapid decline in muscle mass and strength, driven by a complex interplay of reduced protein synthesis, enhanced protein degradation, mitochondrial dysfunction, and impaired neuromuscular signaling [1,13,35–38]. Understanding the physiological consequences of immobilization, particularly in a neutral limb position, as commonly employed in clinical settings is critical for informing both preventive and rehabilitative strategies. On the other hand, immobilization of the foot in plantarflexion or dorsiflexion should be reserved for cases in which such positioning serves a specific clinical objective—for example, to facilitate healing and approximation of the ends of the Achilles tendon following surgical repair. Even in these circumstances, progression toward a neutral position is recommended once the early stages of immobilization have passed.

The integration of infrared thermography, computed tomography, and ultrasound provides a robust framework for non-invasive, longitudinal monitoring of immobilization-induced changes. Each modality captures different aspects of the disuse response, enabling a multifaceted understanding of tissue degradation, moreover, if accompanied by force measurements and changes in morphometric parameters. In the current study, the use of an animal model allowed us to use all of these tools to show the changes that the TA muscle had after cast removal in a neutral immobilization mimicking the muscle atrophy occurring in humans after 7 or 14 days of immobilization.

Classic works support these observations. For example, Spector and colleagues [12] investigated architectural adaptations of the rat soleus (sol), medial gastrocnemius (MG), and TA after 4 weeks of hind limb immobilization using a Plexiglas brace in lengthened, shortened, or neutral positions. Generally, muscles immobilized in a lengthened position preserved mass more effectively than those immobilized neutrally (85% vs. 55% of control) and exhibited fiber elongation alongside greater fiber cross-sectional areas (sol 72%, MG 20% relative to neutral). Neutral immobilization produced moderate atrophy, whereas shortened immobilization resulted in the most severe structural deterioration, with muscle weight falling up to 42% of control and substantial reductions in fiber length. Their findings suggest that the TA is inherently resistant to atrophy during plantar-flexed immobilization, likely due to its unique distal tendon anatomy, which prevents excessive fiber shortening or lengthening during joint fixation. These findings align with our results, where a 15% reduction in TA mass and radial atrophy were observed (Table 1; Fig 1E, F), though the lesser magnitude may be attributed to the shorter immobilization period and the unilateral immobilization used in our protocol.

Further comparison with recent studies supports the translational relevance of our findings. Masiero et. al. [39] employed a custom 3D-printed cast designed to enable non-invasive hind limb immobilization with minimal swelling for the study of disuse-induced skeletal muscle atrophy. Two weeks of immobilization resulted in substantial muscle loss, with gastrocnemius (gast) and soleus (sol) weights reduced by 25% and 31%, respectively, relative to contralateral controls. Correspondingly, muscle fiber cross-sectional area declined by 31% in the gast and 34% in the sol, accompanied by fiber-type–specific alterations. Functional deficits were also evident, as immobilized gast muscles exhibited a 38% reduction in absolute force and a 16% reduction in normalized force. No hypertrophic or overload effects were observed in the contralateral limb, and muscle mass and force were fully recovered within 3 weeks after cast removal. Although the studied muscles are not the same, their outcomes are comparable to those we observed in the TA, suggesting a consistent pattern of immobilization-induced atrophy across different fast-twitch muscles.

Similarly, Brock’s group [38] examined the contractile properties of the plantaris muscle following 14 days of plaster cast immobilization in a neutral position and found a 56% reduction in muscle fiber cross-sectional area and a 49% decline in peak twitch tension, and a markedly increased in the expression of the atrophy-related proteins MAFbx and MuRF1. Brock’s results show markedly greater declines than those observed in our study, a discrepancy that likely reflects inherent muscle-specific differences in susceptibility to disuse. In particular, muscles with higher proportions of slow oxidative fibers or distinct tendon architecture may undergo more pronounced structural and functional deterioration when immobilized, whereas mixed or predominantly fast muscles may exhibit comparatively attenuated losses. These physiological and anatomical distinctions influence responses to reduced loading, activation patterns, and metabolic demand, ultimately shaping the magnitude of atrophy detected across studies.

Human data also corroborate these findings. A systematic review by Campbell et al. [40] reported marked atrophy after short periods of segmental lower or upper limb immobilization in a fixed-angle (neutral) brace model. Pronounced reductions in isometric strength, muscle cross-sectional area reduction (by up to 0.6% per day) during early immobilization, accompanied by a decline in twitch contractility (2% daily) and a reduction in force development (4.4%). Early strength loss exceeds the degree of measurable atrophy, indicating that impairments in contractility in the lower limb and reductions in voluntary activation in the upper limb are major contributors during the initial days of disuse. Although the magnitude of strength loss is comparable between upper and lower limbs, muscle size loss is approximately twice as great in the lower limb. Moreover, immobilization techniques that fix joints in place produce substantially larger deficits in strength and NMF than approaches permitting partial joint movement.

These functional detriments are closely linked to changes in muscle protein turnover, involving well-established molecular pathways of atrophy. Specifically, the ubiquitin–proteasome system, through Atrogin-1/MAFbx and MuRF1 [38], plays a central role in protein degradation. Meanwhile, reduced activation of the IGF-1–AKT–mTOR signaling cascade and increased expression of myostatin contribute to impaired protein synthesis and muscle wasting [1,2,41,42].

The observed reductions in tibialis anterior diameter, muscle volume, and Feret’s fiber diameter in our study are consistent with the hallmark features of radial atrophy. This form of muscle wasting is primarily driven by the loss of myofibrillar content, resulting from an imbalance between protein synthesis and degradation, specifically, a net catabolic state favoring proteolysis [43–45]. In line with this, we also noted a slight increase in the number of centrally nuclei (CNF’s), which is indicative of ongoing degeneration-regeneration cycles. These nuclei are commonly interpreted as markers of muscle fiber turnover and regenerative activity, typically associated with the recovery of muscle fiber size following atrophy [34].

Various imaging approaches have been employed to quantify muscle volume in both clinical and experimental settings. Psatha and collaborators [46] conducted a quantitative 3.0 T MRI analysis of the lower leg in human subjects immobilized in a neutral position due to an ankle fracture. Results revealed progressive muscle deterioration during 6 weeks of lower-leg immobilization, with total muscle volume declining by 17%. The greatest CSA loss occurred in the gastrocnemius medialis (23%), followed by the soleus (19%), gastrocnemius lateralis (17%), and TA (10%), with measurable CSA reductions also present in the contralateral limb. Authors also found increases in T_2_, relaxation times across all muscles, indicating biochemical and structural alterations complemented with a significantly reduction in the pennation angle in the immobilized leg. In comparison, we observed a more pronounced reduction in TA volume of approximately 40% (see Table 1). This discrepancy may be attributed to differences in methodology, *in vivo* MRI performed in humans versus our assessments in a rat model.

Similarly, Wall’s group [47] investigated the effects of immobilization on the quadriceps muscle, a fast-twitch group, comparable in fiber type to the TA using CT, dual-energy X-ray absorptiometry (DEXA), and MRI. They found that quadriceps muscle disuse for 5 or 14 days induced rapid and substantial atrophy, with cross-sectional area declining by 3.5% and 8.4%, respectively. Strength loss was disproportionately larger, decreasing by 9.0% after 5 days and 22.9% after 14 days, while leg lean mass declined by 1.4% and 3.1%. Immobilization triggered a marked catabolic response, as myostatin mRNA doubled in both groups and MAFbx mRNA increased at both time points. MuRF1 mRNA rose significantly only after 5 days, indicating an early transcriptional surge in proteolytic signaling. Although our model involved a different species and muscle, the trend in functional decline is consistent; we observed a strength reduction of approximately 50% compared to control, which mirrors the relative temporal dynamics of atrophy-induced weakness described in human studies.

It is well-established that limb immobilization, leads to rapid strength loss driven by both decreased muscle mass and reduced force-generating capacity per unit of CSA. This functional impairment is further exacerbated by metabolic alterations, including reduced glycogen and ATP content at rest, accelerated depletion during muscle activity, increased lactate accumulation, and diminished fatty acid oxidation [1]. These physiological disruptions collectively contribute to increased muscle fatigability and highlight the broad systemic impact of disuse atrophy.

Infrared thermography (IR) is a rapid, non-invasive method for assessing the radiative temperature of the skin surface. In the context of musculoskeletal injury or disuse, increased blood flow to the affected area facilitates the delivery of proteins and immune cells necessary for tissue repair. This vascular response leads to vasodilation, local redness, and elevated surface temperature, signs of inflammation [15,48–50]. Moreover, muscle regeneration processes including the activation of satellite cells responsible for myofiber repair also contribute to the global temperature increment [6,50].

Inflammation is characterized by the release of prostaglandins, cytokines, and other mediators that not only facilitate tissue repair but also promote vascular changes that increase blood flow and heat in the affected region. These physiological processes can lead to localized temperature elevations in muscles such as the tibialis anterior (TA), particularly in regions undergoing structural or metabolic stress. IR imaging captured temperature profiles of the entire hind limb after cast removal. Our data revealed elevated temperatures at 14 days of immobilization (Table 1, Fig 1 A–C), which we interpret as heightened inflammatory activity and muscle stress resulting from prolonged disuse. These observations align with prior studies that demonstrated IR imaging as a valuable tool for detecting inflammation through thermal signatures in target tissues [51].

Computed tomography (CT) has long been considered a reliable imaging modality for skeletal muscle visualization, particularly when enhanced with contrast agents to overcome the low density of soft tissues relative to bone. In this study, we employed ethanol as an alternative to contrast agents to enhance soft tissue contrast. Protein denaturation occurs during alcohol-based fixation due to dehydration of functional groups [26,28]. Because the same fixation protocol was applied uniformly across all samples (C and Imm), dehydration-related shrinkage was comparable across groups.

Our CT analysis revealed a progressive increase in dark striated regions within the muscle at 7 and 14 days (10% and 22%, respectively), which we attribute to air, fatty infiltration, connective tissue accumulation, or residual perimuscular adipose tissue [35]. These features corresponded with the observed reductions in muscle size and volume (Table 1). To date, only a limited number of studies have applied CT imaging to investigate skeletal muscle disuse atrophy [24,52], reporting comparable declines in muscle mass in regions such as the thigh and TA.

Ultrasound imaging further complements IR and CT by offering high spatial resolution and detailed visualization of muscle architecture. This is a practical, non-invasive technique to assess muscle morphology, including thickness, pennation angle, connective tissue organization, and pathological changes such as fibrosis, myositis ossificans, or inflammation [10,53]. Narici and Carretelli [54] observed a reduction in pennation angle and cross-sectional area (CSA) of the medial gastrocnemius in patients with unilateral lower limb atrophy. While our ultrasound findings similarly indicated a decrease in CSA at 7 and 14 days (Fig 1D–F), we did not detect any significant changes in pennation angle (Fig 1G–I). This may be due to the limited imaging area or the relatively short duration of immobilization.

In a related study, Mayer et al. (2020) [55] investigated the effects of early skeletal muscle deterioration in the ICU after 7 days of disuse in human subjects. Rectus femoris cross-sectional area declined markedly by a median of 18%, accompanied by a 10% rise in echointensity, indicating rapid structural degradation. Although our ultrasound data were qualitative, changes in echointensity were evident and aligned with trends reported in the literature. Quantitative assessment of CSA was primarily achieved through CT analysis in our study, while echotexture changes, were perceptible and supported by US.

According to existing literature, the tibialis anterior is predominantly classified as a fast glycolytic muscle, with its fiber-type composition comprising approximately 4–5% slow-twitch fibers and 95% fast-twitch fibers. Of the latter, roughly half are fast oxidative (intermediate) and the remaining half fast glycolytic [56]. Following a period of immobilization in the neutral position, we observed a shift in fiber-type composition characterized by an increase in intermediate fibers and a concomitant decrease in fast glycolytic fibers (Table 1, Fig 3C–H, K). Furthermore, muscles immobilized for 7 and 14 days showed a significant decrease in specific force (Fig 2A–C), as well as a significant increment in time fatigue with continuous stimulation (Fig 2 D), and no change in fatigue time during repeated short tetany (Fig 2E, F). These findings agree with previous reports on TA muscle fiber types and immobilization-induced remodeling [57]. Hortobágyi group [57] examined 48 men and women following 3 weeks of knee immobilization and after 12 weeks of retraining with 1866 eccentric, concentric or mixed contractions founding that after this Imm period in a neutral joint position reduced eccentric, concentric, and isometric strength of the quadriceps by 47% after two weeks of spontaneous recovery. Muscle fiber cross-sectional areas of type I, IIa, and IIx fibers declined by 13%, 10%, and 10%, respectively, and remained approximately 5% smaller than baseline after recovery. These findings highlight the critical influence of muscle length and contraction mode on neuromuscular adaptation following immobilization at neutral joint angles.

To the best of our knowledge, this is the first study to integrate three clinical imaging modalities—infrared thermography (IR), computed tomography (CT), and ultrasound (US)—alongside force measurement and histological analysis to evaluate the effects of neutral position immobilization on the TA muscle. Further quantitative investigations are currently underway, including assessments of protein expression and atrogene levels, aimed at elucidating the molecular mechanisms of disuse induced muscle remodeling after immobilization in neutral position. Future studies will also incorporate contrast agents for CT to improve muscle visualization, as well as the application of the Heckmatt Scale to quantify echogenicity and better characterize ultrasound changes in both fast- and slow-twitch muscles.

Employing a multimodal approach to investigate hind limb immobilization in a neutral position bridges critical gaps between structure, function, and pathological outcomes. This strategy enhances the translational reliability of rodent models, deepens our understanding of disuse-related conditions including sarcopenia, joint contractures, and neurovascular impairments.

Ultimately, this knowledge informs the development of targeted rehabilitation protocols and therapeutic interventions aimed at mitigating the adverse effects of immobilization in clinical populations after an immobilization.

## Supporting information

S1 Table Twitch and tetany for control, 7 and 14 days of Immobilization (n = 3). (TIF)

S2 Table Relationship between specific force (N/cm2) and frequency (Hz) of stimulation from single pulse, summation to tetanus for TA muscle at three different time-points (n = 3). (TIF)

S3 Table Fatigue induced by the continuous stimulation protocol and repeated short-tetany for three animals at different time points. (TIF)
