# Supplementary figures and images for: Multimodal imaging of tibialis anterior muscle adaptations to neutral-position immobilization
Source: PLoS One. 2026 Jan 30;21(1):e0339510. doi: 10.1371/journal.pone.0339510 (PMC12857925; doi:10.1371/journal.pone.0339510)

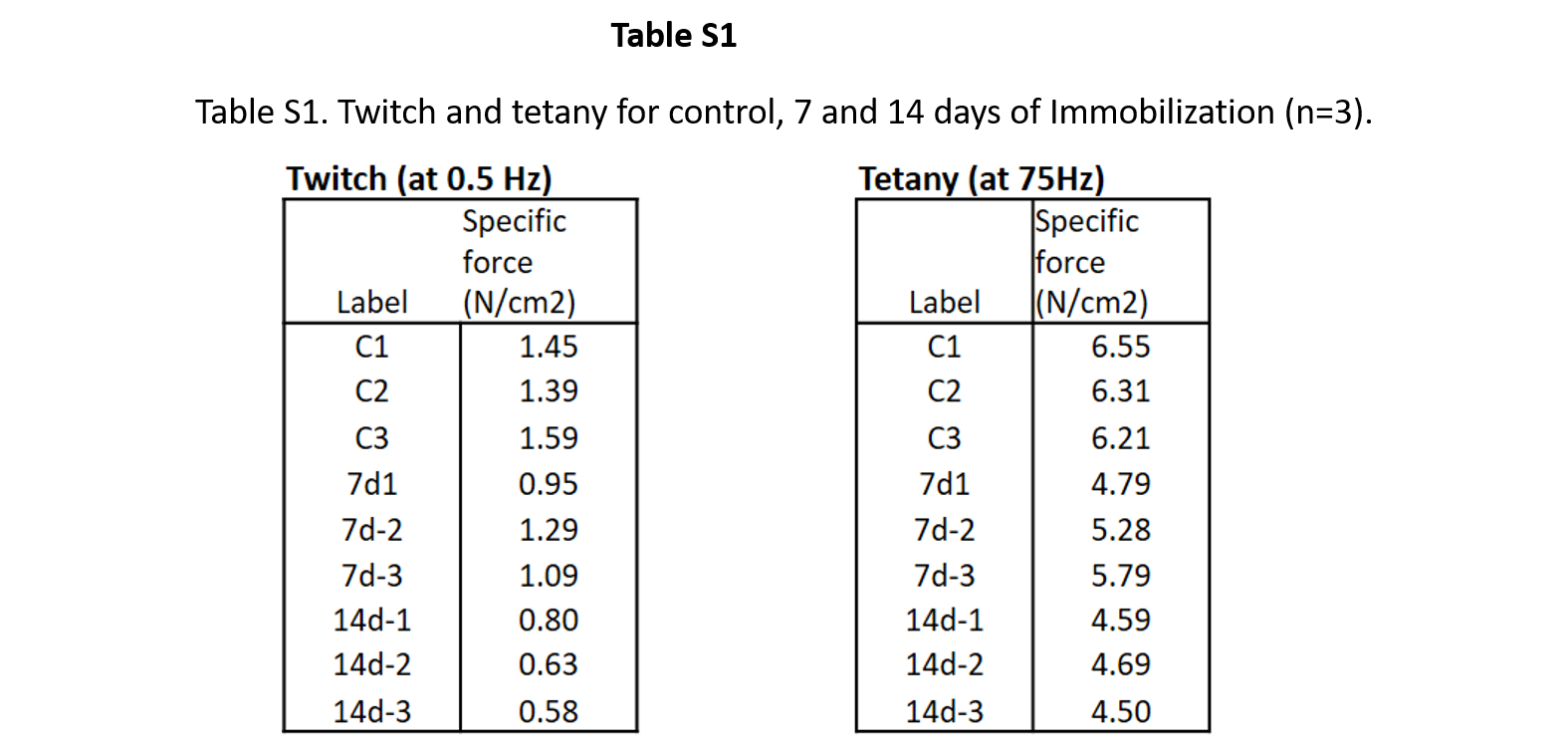

Supplement: S1 Table — (TIF) [file pone.0339510.s001.tif]

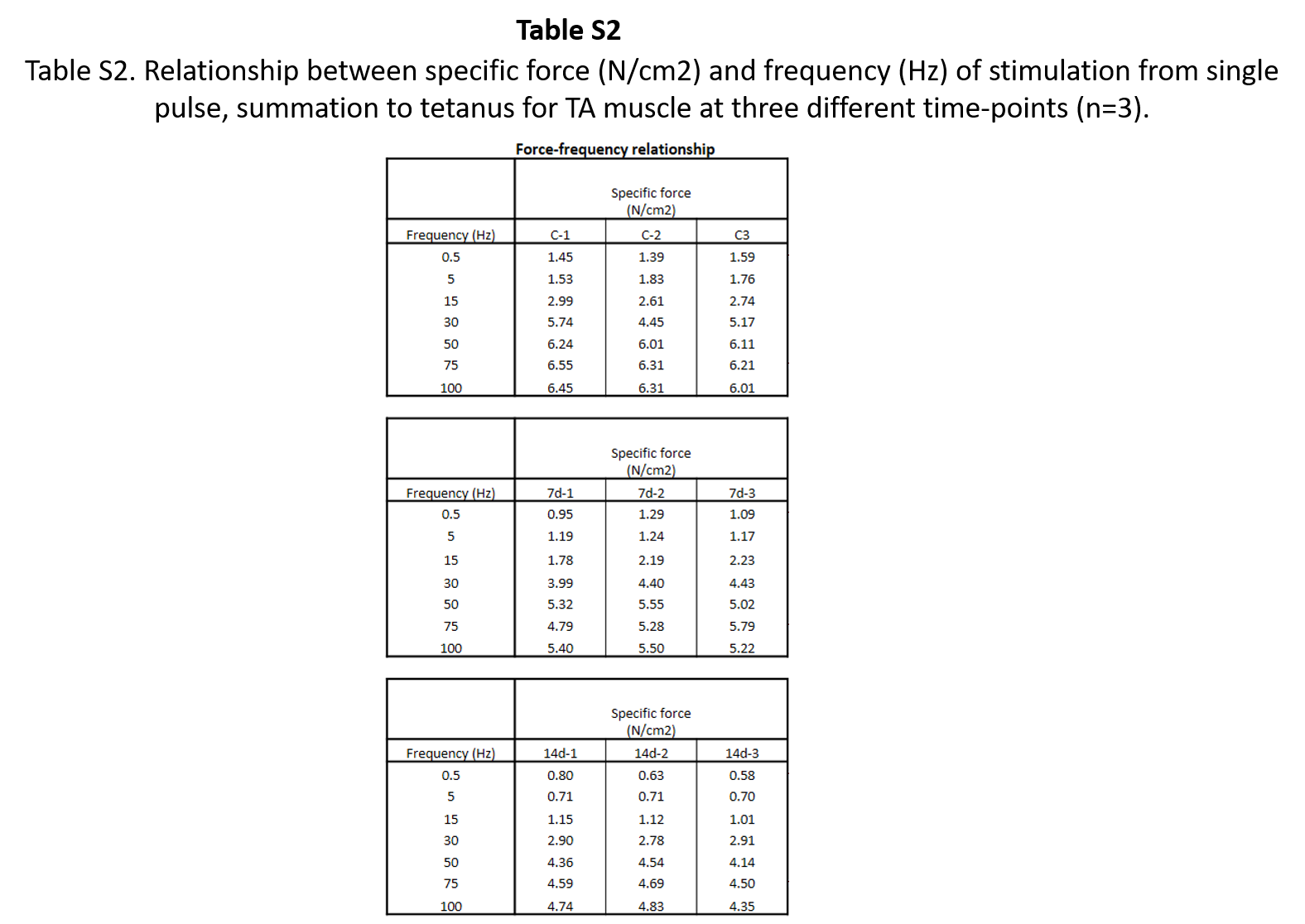

Supplement: S2 Table — (TIF) [file pone.0339510.s002.tif]

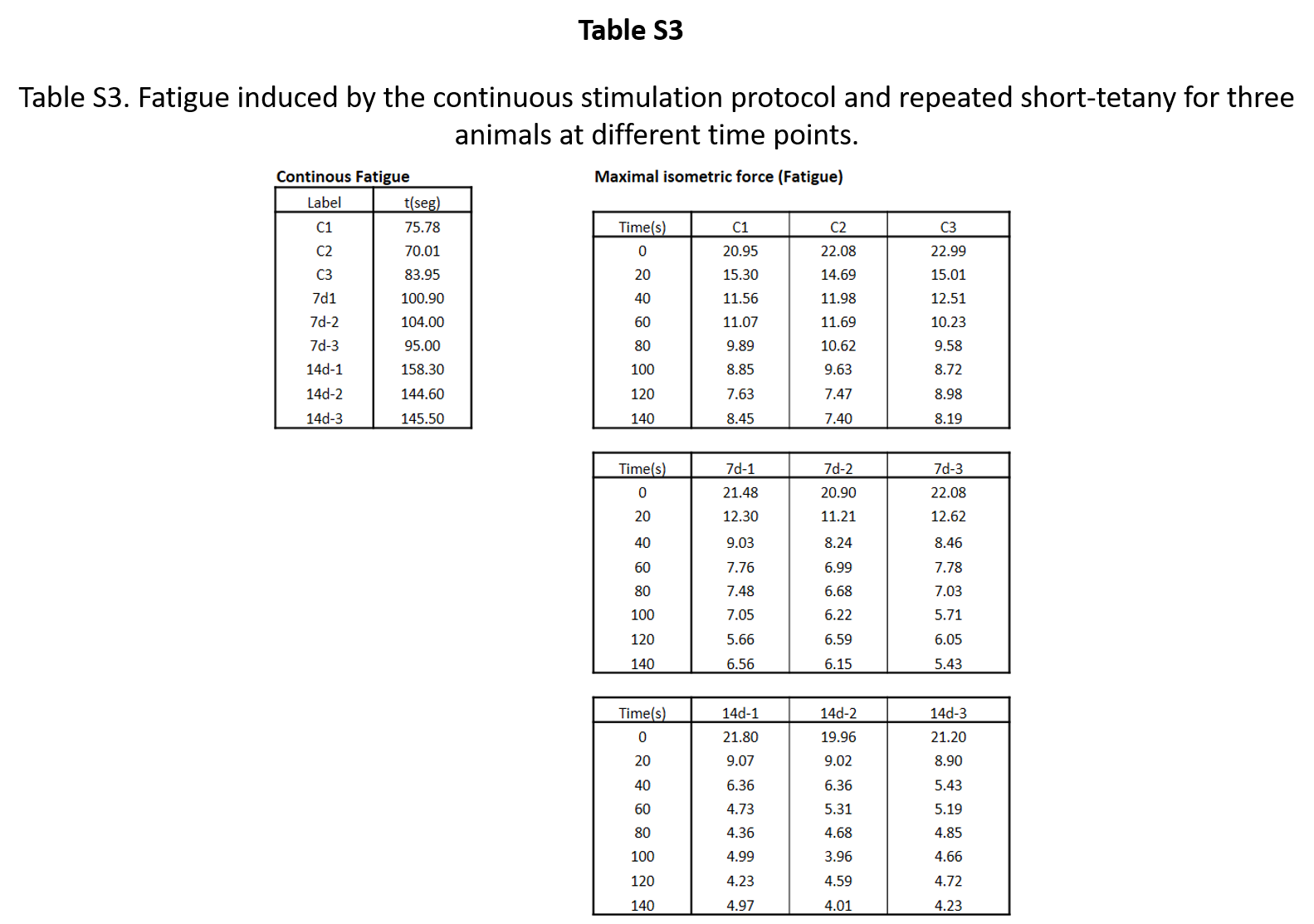

Supplement: S3 Table — (TIF) [file pone.0339510.s003.tif]
